# Prevalence and risk factors of urogenital schistosomiasis among under-fives in Mtama District in the Lindi region of Tanzania

**DOI:** 10.1371/journal.pntd.0010381

**Published:** 2022-04-20

**Authors:** Vivian Mushi, Abdallah Zacharia, Magdalena Shao, Marycelina Mubi, Donath Tarimo

**Affiliations:** 1 Department of Parasitology and Medical Entomology, School of Public Health and Social Sciences, Muhimbili University of Health and Allied Sciences, Dar es Salaam, Tanzania; 2 Department of Environmental and Occupational Health, School of Public Health and Social Sciences, Muhimbili University of Health and Allied Sciences, Dar es Salaam, Tanzania; Natural History Museum, UNITED KINGDOM

## Abstract

**Introduction:**

Despite the ongoing intervention for schistosomiasis control among school-age children in the Lindi region of Tanzania, urogenital schistosomiasis continues to be a public health problem, presumably because other at-risk populations are not covered in praziquantel deworming campaigns. Evidence shows that under-fives become infected in their early life hence the need to understand the disease profile and the risk factors for exposure to infection so as to plan effective control strategies in this group. This study examined the prevalence and risk factors of urogenital schistosomiasis among under-fives in the Mtama district, Lindi region of Tanzania.

**Methodology/Principal findings:**

A quantitative community-based cross-sectional study was carried out among 770 participants (385 under-fives and their 385 parents/guardians) in the Mtama district to investigate the burden and the risk factors associated with *S*. *haematobium* infection. A single urine specimen was collected from the under-fives and tested for macro and microhaematuria, presence of *S*. *haematobium* ova, and intensity of infection. A structured questionnaire gathered on risk factors for *S*. *haematobium* exposure in under-fives from their parents/guardians. Data analysis was performed using descriptive statistics, chi-square test, and logistic regression.

Prevalence of *S*. *haematobium* ova was 16.9%, and that of macro and microhaematuria was 6% and 17.9%, respectively. Of the 65 positive under-fives, 49 (75.4%) 95% CI 65.4–86.3 had a light infection intensity, and 16 (24.6%) 95% CI 13.7–35.5 had a heavy infection intensity. Among the assessed risk factors, the parents/guardians habit of visiting water bodies for domestic routines (AOR: 1.44, 95% CI: 1.13–1.74), especially the river (AOR: 6.00, 95% CI: 1.20–35.12), was found to be a significant risk factor for infection of *S*. *haematobium* in under-fives.

**Conclusion/Significance:**

A moderate prevalence of *S*. *haematobium* was found among the under-fives conceivably with adverse health events. The infected under-fives could be a source of continuity for transmission in the community. An intervention that covers this group is necessary and should be complemented with regular screening, health education campaigns, and an adequate supply of safe water.

## Author summary

Urogenital schistosomiasis caused by *S*. *haematobium* has been highly endemic in Lindi-Tanzania for more than three decades. Due to the burden of the disease, preventive chemotherapy with praziquantel was put in place to control the infection. However, this intervention has focused merely on school-aged children, excluding preschool-age children, despite the evidence of early exposure to the infested water. A community-based cross-sectional study was conducted to investigate the current burden and the risk factors of urogenital schistosomiasis among the under-fives of Mtama district, Lindi region. The prevalence of urogenital schistosomiasis in under-fives was 16.9% with approximately a quarter (24.6%) being heavily infected. The factors responsible for the ongoing transmission of urogenital schistosomiasis in under-fives were the parental/guardians’ inadequate knowledge (31.1%), coupled with negative attitudes (40.5%), and inappropriate practices (34.3%) regarding the disease transmission, treatment, and prevention. Also, inadequate supply of safe water and sanitation facilities, unhygienic practices, and intense parental/guardian water contact activities. The parents/guardians’ practices of visiting the water bodies, especially the river, with the under-fives were significantly associated with the early acquisition of the disease. Hence, there is a need for regular monitoring of under-fives, provision of health education to parents/guardians, and adequate provision of safe water.

## Introduction

Urogenital schistosomiasis caused by *Schistosoma haematobium* is among the water-borne neglected tropical diseases associated with significant morbidity and mortality in tropical and subtropical areas [1,2]. Approximately 436 million people in 78 endemic countries are at risk of acquiring urogenital schistosomiasis, and over 112 million people are infected [1]. *S*. *haematobium* requires freshwater snails (*Bulinus* species) for the development of the infective stage of the parasite (cercariae), which subsequently infects humans who come into contact with the water during domestic routines, recreational activities, and occupation [1,3,4]. Preschool-aged children (PSAC), school-aged children (SAC), and people involved in water-related occupations are at high risk of infection, especially in communities with inadequate supply of clean and safe water, poor sanitation, and unhygienic practices [1,2].

Under-fives are often exposed to infested water when they accompany their parents/guardians or when they are bathed with the infested water [5–7]. Evidence shows that the under-fives can acquire infection in their early life leading to poor growth, poor cognitive function, iron-deficiency anemia, malnutrition, development of hepatosplenic morbidities, and reduced school performance [8]. The ongoing control of urogenital schistosomiasis in endemic areas is mainly through the Mass Drug Administration (MDA) of praziquantel preventive chemotherapy [9]. However, historically, preventive chemotherapy has primarily focused on SAC, excluding PSAC due to lack of suitable drug formulation together with a lack of treatment and prevention policies for this age group [8,10].

Tanzania is ranked second in terms of the burden of schistosomiasis in sub-Saharan Africa, with different levels of endemicity across the country [11–13]. Urogenital schistosomiasis has been extensively studied in SAC in some of the regions of Tanzania, neglecting PSAC due to limited evidence of early exposure to infection [14]. Lindi region is among the high-risk communities with a history of a high prevalence of urogenital schistosomiasis (above 50%) in SAC [15]. This high burden of urogenital schistosomiasis is linked to the abundance of suitable intermediate snail hosts (*Bulinus* species) in the area, inadequate supply of clean and safe water, and inadequate sanitation facilities. Lindi has the highest proportions (68.3%) of households without improved forms of toilets and ranks the second region in Tanzania with the highest proportions of households (6.6%) without any form of toilet [16]. In addition, Lindi has a scarcity of clean and safe water, with only 40.1% having access to tap water [17]. The majority of the communities use unprotected water sources putting them at risk of urogenital schistosomiasis.

With the high burden of urogenital schistosomiasis in the Lindi region, PSAC have been overlooked, despite the evidence from other endemic areas showing that they carry a significant level of infection and contribute to transmission [14,18]. This study investigated the burden and the risk factors for urogenital schistosomiasis among under-fives in the Mtama district in the Lindi region of Tanzania. The data will assist the Tanzania Neglected Tropical Diseases Control Programme in the planning of the prevention strategies for the under-five population, reducing associated morbidity and overall urogenital schistosomiasis transmission.

## Materials and methods

### Ethics statement

Ethical clearance was obtained from the Muhimbili University of Health and Allied Sciences (MUHAS) Ethical Review Board (MUHAS-REC-12-2020-457). Permission to conduct the study in the Mtama district was obtained from the required administrative units in the Lindi region and Mtama district. Before the commencement of the data collection, the researcher and research assistants explained to the parents/guardians of the under-fives the objectives of the study and requested consent. The parents/guardians who agreed with their children to participate in the study signed informed consent forms. The results were communicated to the parents/guardians and positive children were treated free.

### Study area and demographics

Mtama District Council (DC), is one of the six district councils of the Lindi region and lies at latitude 10° 18’ 0" S and longitude 39° 22’ 0" E. The district council is bordered to the North, South, East, and West by Kilwa DC, Mtwara region, the Indian Ocean and Lindi Municipal Council, and Nachingwea DC, respectively. The district has 31 wards with an approximate population of 194,143 (91,647 males and 102,496 females) [19].

The environment of the Mtama district (annual average rainfall of 910 mm and an average temperature of 26.3°C) favors the breeding and survival of *Bulinus* snails (the intermediate host of *S*. *haematobium*) [15]. The community members of the Mtama district are engaged in agriculture, livestock keeping, and fishing. Available water sources include taps, rivers, springs, dams, irrigational schemes, dug wells, and ponds. Mtama was selected because of the history of a high prevalence of urogenital schistosomiasis in school-aged children (58.9%) [15,20].

### Study design and population

A community-based cross-sectional study using a quantitative method of data collection was carried out in Mtama DC from April to May 2021 to investigate the prevalence and risk factors of urogenital schistosomiasis in under-fives. The study population was under-fives for *S*. *haematobium* infection and their parents/guardians for the risk factors of urogenital schistosomiasis among the under-fives.

### Sample size determination and sampling

The sample size of the study participants was estimated using the formula for cross-sectional surveys (n = z^2^ P (100-P)/ ε^2^) [21], set for a prevalence of 50% due to lack of previous data from the area, standard normal deviate of 1.96 on using 95% confidence interval, and margin of error (5%). In addition, the sample size was adjusted for a 10% non-response rate and designing effect of 1.5. Hence, the total sample size was 634 under-fives with their associated parents/guardians.

**Fig 1:**
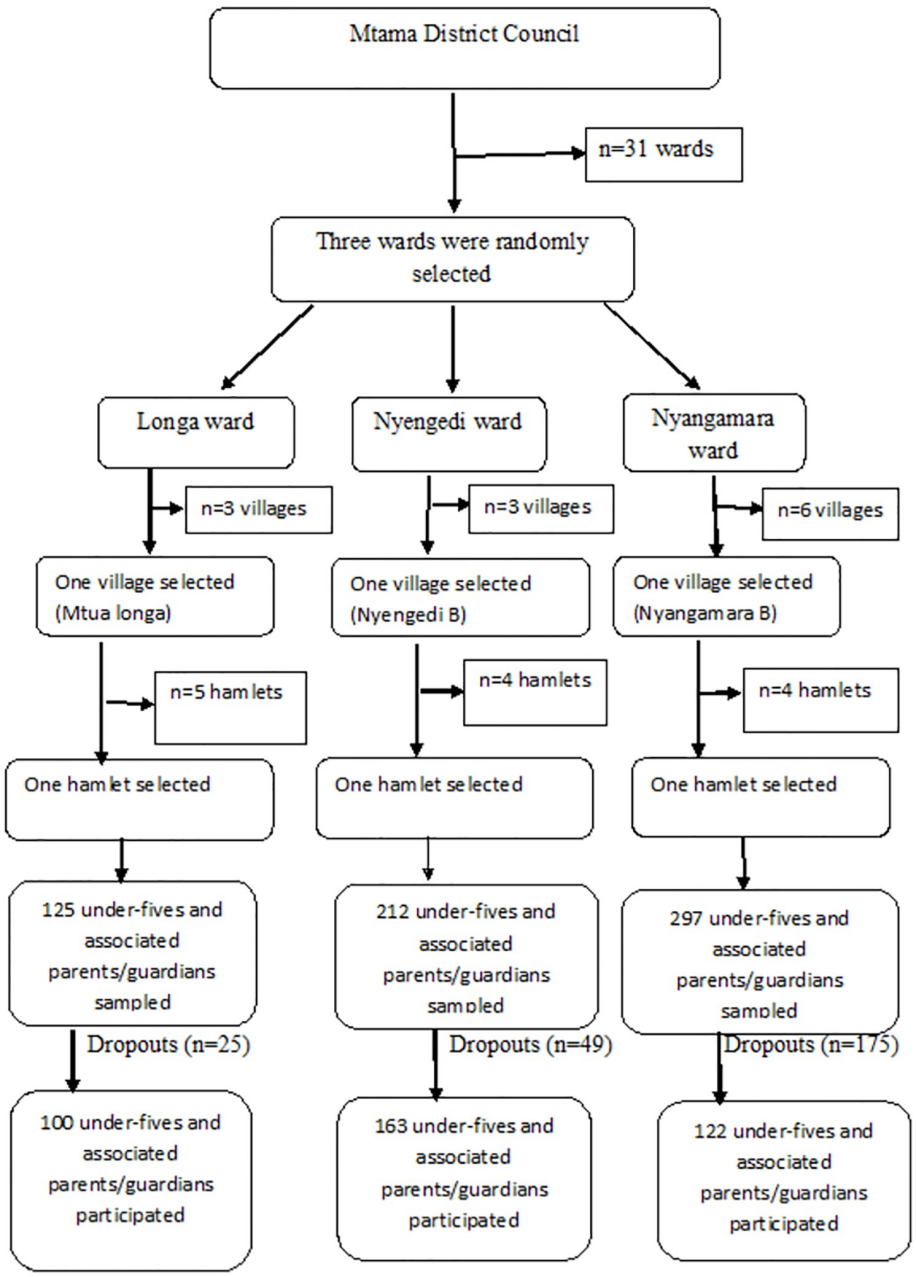
Flow chart of sampling technique.

A three-stage cluster sampling technique was employed to recruit 634 under-fives and their associated parents/guardians as described in Fig 1, whereby the first stage involved a simple random selection of three endemic wards from the list of 31 urogenital schistosomiasis endemic wards in Mtama District. In the second stage, a single village was randomly selected per selected ward, and in the third stage, from each selected village, a single hamlet was randomly selected. This resulted in the selection of three hamlets for inclusion in the study. In each of the randomly selected hamlets, the executive officers were requested to provide a list of all households which contained at least one under-five. Unfortunately, in all wards, the list wasn’t available. Therefore, the population of each ward was used to determine the number of under-fives sampled in each hamlet. The randomly selected wards (Longa, Nyengedi, and Nyangamara) had a population of 3185, 5390, and 7545, respectively. The estimated population from each selected ward was used to calculate the number of under-fives sampled per ward to obtain the required total sample size. As a result, a total of 125, 212, and 297 under-fives and their associated parents/guardians were sampled from Mtua-longa, Nyengedi B, and Nyangamara hamlets, respectively. However, only 100, 163, and 122 parents/ guardians agreed and signed informed consent for their children to participate in the study. In the case of households with more than one under-fives, one child only was randomly selected. Therefore, the under-fives were the target population for inference. The sample size of the recruited under-fives and their parents/guardians was smaller (385) compared to the estimated sample size (634) due to either the rejection of the parents/guardians to participate, failure of the parents/guardians to provide the child urine or participating in the questionnaire survey.

### Sample collection and processing

A single urine sample was collected from each under-five, and each associated parent/guardian was interviewed. The parents/guardians were given a labeled container/urine collection bag with ID numbers for urine collection of their children and instructed to collect about 30 milliliters of fresh urine between 10:00 to 14:00 hours which is the optimum time for egg shedding. The urine samples were firstly examined macroscopically by their color for visible haematuria and then followed by the detection of microhaematuria using urine reagent strips (dipstick) (Mission Urinalysis Strips, USA). The presence of *S*. *haematobium* ova in each urine sample was identified using the urine filtration technique [22]. All urine samples were shaken then 10 ml of each urine sample was drawn into a plastic syringe and pushed through a polycarbonate filter with a pore size of 12 μm (Costar Corporation, USA). All urine filters were placed on the microscope slides, followed by a drop of Lugol’s iodine to stain the *S*. *haematobium* ova, and then the slides were examined at X40 magnification under a microscope by an experienced laboratory scientist [22]. The presence and number of *S*. *haematobium* ova on each filter were recorded on a laboratory urine analysis form as described in S1 File.

### Questionnaire survey

A pre-tested structured questionnaire assessed the risk factors for *S*. *haematobium* infection from each under-fives parents/guardians. The questionnaire had five sections (A-E). Section A collected socio-demographic data of the under-fives and their parents/guardians. Section B assessed the parental/guardian’s knowledge on urogenital schistosomiasis, while section C assessed the WaSH practices of the under-fives and their parents/guardians. Sections D and E collected information on the attitudes and practices, respectively, of the parents and guardians towards urogenital schistosomiasis. The questionnaire was administered to interviewees by trained research assistants. An English and Kiswahili version of the questionnaire is supplied as S2 and S3 Files, respectively.

### Quality control

The questionnaire was pre-tested at Makonde village in the Mtama district. The Makonde village used in pretesting the questionnaire was excluded from data collection. The pre-testing involved 32 parents/guardians (5% of the calculated sample size). Thirty-nine urine samples (10%) examined for *S*. *haematobium* ova were sent to another laboratory scientist for re-examination and comparisons. The *S*. *haematobium* eggs were prevented from hatching before sending the samples to another laboratory by adding 2–3 drops of formalin in each container containing urine to fix the eggs and preserve the urine. The results obtained from the quality control laboratory on the presence or absence of *S*. *haematobium* matched with ours. However, there was a variation in the intensity whereby the number of counted eggs was either a little high or low compared to our observation (S4 File).

### Data analysis

Data collected was entered and analyzed using Statistical Package for the Social Sciences (SPSS) version 23 (IBM Corp., Armonk, NY, USA). Descriptive statistics were used to summarize the frequency and proportions of independent and dependent variables. Prevalence of urogenital schistosomiasis, macro and microhaematuria were further summarized based on the socio-demographic characteristics of the under-fives. Intensity of infection was categorized as 1–50 eggs/10 mls (light infection intensity), and > 51 eggs/10 mls (heavy infection intensity). The Pearson’s chi-square test and their p-value were used to compare the proportions between groups. Univariate and multivariate logistic regression was used to assess the predictors for urogenital schistosomiasis in the under-fives. All the independent variables with a p-value < 0.25 were subjected to multivariate analysis. The outcome variable used in regression analysis was the prevalence of *S*. *haematobium*.

Knowledge was tested using eight questions with 14 responses and analyzed using a scoring scale. The responses were scored one mark for correct and zero for incorrect, and then the total score was calculated for each participant to classify their knowledge levels. The scores were classified as follows; ≤ 4 as a low level of knowledge, 5–9 as a moderate level of knowledge, and ≥ 10 as a high level of knowledge. A five-point Likert scale was used to assess attitudes and practices of the parents/guardians on urogenital schistosomiasis using ten statements per section. The reliability analysis for the attitude and practice statements yielded Cronbach’s alpha values of 0.716 and 0.798, respectively. The sum scores for attitude and practice sections were calculated for each participant, ranging from 10–50 points. Then the mean attitude score (38) and practice score (36) was used to classify the levels of attitude and practice. The scores of ≤ 37 and ≥ 38 were classified as negative and positive attitudes, respectively. Similarly, the scores of ≤ 35 and ≥ 36 were categorized as inappropriate and appropriate practices, respectively.

### Distribution of praziquantel to positive under-fives

The praziquantel at the recommended dose of 40 mg/kg of body weight was used to treat under-fives found positive for *S*. *haematobium* eggs. The appropriate dose for each under-five was determined by measuring the child’s weight. Then the parents/guardians were instructed to feed the children before administering the praziquantel. Also, the local pharmacist advised the parents/guardians to crush the drug and dissolve it in juice or water for easy swallowing of the praziquantel dose.

### Operational definitions of key terms

Urogenital schistosomiasis refers to the presence of *S*. *haematobium* ova in the examined urine. Infection intensity refers to the number of eggs/ova observed per 10 mls of urine. Under-fives refer to children less than five years old, classified as young (12–35 months) and preschoolers (36–59 months). A representative village/hamlet refers to a selected village/hamlet in each of the selected wards reflecting the characteristics of the remaining villages/hamlets.

## Results

### Socio-demographic characteristics of the under-fives and their parents/guardians

This study recruited 770 participants (385 under-fives and their 385 parents/guardians). The response rate was 60.73%. More than half of under-fives were aged 12–35 months (52.5%) and females (53.5%), respectively. More than two-thirds of the parents/ guardians (69.1%) were aged 18–35 years with a mean age of 31.05 years. More than half of the parents/guardians (61.3% and 60.9%) had attained primary education and were residents of the Mtama district for 18–35 years, respectively (Table 1).

**Table 1:** Socio-demographic characteristics of the under-fives and their parents/guardians (n = 770).

Variable	n (%)	95% CI
**Sex of the children**		
Males	179(46.5)	41–51.3
Females	206(53.5)	48.7–59
**Age (months) of the children**	**33.87 ± 14.7**	
12–35 (Young children)	202(52.5)	47.2–58.2
36–59 (Pre-schoolers)	183(47.5)	41.8–52.8
**Sex of the parents/guardians**		
Males	30(7.8)	5.1–10.7
Females	355(92.2)	89.3–94.9
**Age (years) of the parents/guardians**	**31.05 ± 10.17**	12.6–18.2
12–17 (Teen)	10(2.6)	1.0–4.5
18–35 (Young adults)	266(69.1)	64.9–74.3
36–55 (Middle aged adults)	99(25.7)	20.8–29.6
> 56 (Older)	10(2.6)	1.3–4.2
**Education level of the parents/guardians**		
Never attended school	110(28.6)	24.3–32.8
Primary school	236(61.3)	56.8–66.3
Secondary school	35(9.1)	6.2–12
Post-secondary training	1(0.3)	0.0–1.0
University	3(0.8)	0.0–1.6
**Marital status of the parents/guardians**		
Married	257(66.8)	61.3–71.4
Single	99(25.7)	21.3–30.1
Divorced	24(6.2)	3.9–8.9
Cohabiting	2(0.5)	0.0–1.3
Widow(er)	3(0.8)	0.0–1.8
**Occupation of the parents/guardians**		
Housewife	23(6.0)	3.6–8.1
Peasant*	324(84.2)	80.8–87.8
Petty business**	32(8.3)	5.4–11.2
Employed	6(1.6)	0.4–2.9
**Residency (years) of the parents/guardians**	**26.40 ± 12.8**	
≤ 11	55 (14.3)	11.2–17.9
12–17	17 (4.4)	2.3–6.8
18–35	234(60.9)	56.2–65.5
36–55	71 (18.4)	14.0–22.3
> 56	8(2.1)	0.8–3.6
**Wards of the residency**		
Longa	100(26)	22.1–30.7
Nyengedi	163(42.3)	37.6–47.3
Nyangamara	122 (31.7)	27.5–36.6

*Peasant refers to small scale farmer

** Petty business refers to a small-scale business with less capital investment

### Prevalence of urogenital schistosomiasis, macro and microhaematuria, and infection intensity of the study participants

The overall prevalence of urogenital schistosomiasis (egg positive), macro and microhaematuria was 16.9%, 6%, and 17.9%, respectively. The prevalence of urogenital schistosomiasis was higher among under-five males (18.4%), the PSAC aged 36–59 months (17.5%), and the under-fives of the Longa ward (43%). There was a statistically significant difference in the prevalence of urogenital schistosomiasis between the wards (p < 0.000) (Table 2).

**Table 2:** Prevalence of urogenital schistosomiasis by urine microscopy, macro and microhaematuria stratified according to socio-demographic characteristics of the study participants (n = 385).

Socio-demographics	Total	*S*. *haematobium* prevalence n(%)	p-value	Prevalence of macro haematuria n(%)	p-value	Prevalence of micro haematuria n(%)	p-value
**Sex of the children**							
Males	179	33(18.4)	0.448	11(6.1)	0.895	32(17.9)	0.983
Females	206	32(15.5)		12(5.8)		37(18)	
**Age (months) of the children**							
12–35 (Young children)	202	33(16.3)	0.764	14(6.9)	0.405	36(17.8)	0.957
36–59 (Preschoolers)	183	32(17.5)		9(4.9)		33(18)	
**Wards of the residency**							
Longa	100	43(43)	0.000*	7(7)	0.718	38(38)	0.000*
Nyengedi	163	13(7.9)		8(4.8)		22(13.3)	
Nyangamara	122	9(7.5)		8(6.7)		9(7.5)	
**Total**	**385**	**65(16.9)**		**23(6)**		**69(17.9)**	

*Statistically significant (p < 0.05)

Based on the WHO criteria for the categorization of infection intensity [23]; of the 65 under-fives who were egg positive for urogenital schistosomiasis, 49 (75.4%, 95% CI 65.4–86.3) had light infections and 16 (24.6%, 95% CI 13.7–35.5) had heavy infections. The mean (SD) number of eggs/10 ml of urine was 48.42 (99.14) and ranged from 1 to 523. The under-fives males (36.4%) had higher infection intensity compared to under-fives females (12.5%), and the observed difference was statistically significant (p = 0.026) (S1 Table).

### Knowledge on urogenital schistosomiasis among the parents/guardians of the under-fives

The majority of the parents/guardians (98.7%) had heard of urogenital schistosomiasis, with the most mentioned source of information being the health centers (63.7%). Nearly two-thirds of the participants (61.6%) correctly knew that urogenital schistosomiasis infection was acquired through contacting infested water. However, only 11.3% understood that worms were the cause of urogenital schistosomiasis. The majority (88.4% and 81.3%) knew that urogenital schistosomiasis was curable and preventable, respectively, with the most mentioned prevention being the use of anti-schistosomal medicine (27.5%) (Table 3).

**Table 3:** Knowledge on urogenital schistosomiasis among the parents/guardians of the under-fives (n = 385).

Variable	n (%)	95% CI
**Heard of urogenital schistosomiasis**		
Yes	380(98.7)	97.4–99.7
No	5(1.3)	0.3–2.6
**Source of the information**		
Health center	242(63.7)	59.3–68.3
Mass media	24(6.3)	3.7–8.6
Community health worker	43(11.3)	7.9–14.2
Friend	47(12.4)	9.2–15.7
Street	18(4.7)	2.6–7.1
School	6(1.6)	0.5–2.9
**Causative agent**		
Bacteria	65(17.1)	13.3–21.0
Virus	6(1.6)	0.5–2.9
Parasite	43(11.3)	8.0–14.8
Fungi	2(0.5)	0.0–1.3
Do not know	264(69.5)	64.4–74.2
**Mode of urogenital schistosomiasis transmission**		
By contacting infested water	234(61.6)	56.1–66.6
By eating contaminated food	12(3.2)	1.7–5.0
Through sexual intercourse	4(1.1)	0.3–2.1
By drinking dirty water	19 (5)	2.9–7.6
Do not know	111 (29.2)	24.7–33.4
**Do snails transmit urogenital schistosomiasis**		
Yes	173 (45.5)	40.2–50.8
No	24 (6.3)	3.8–8.7
Do not know	183(48.2)	43.2–53.4
**Symptoms of urogenital schistosomiasis**		
Blood in the urine	229 (60.3)	54.9–66.3
Dysuria	15 (3.9)	2.1–6.0
Stomachache	6(1.6)	0.5–2.9
Diarrhea	1(0.3)	0.0–0.8
Headache	2 (0.5)	0.0–1.3
Itching of genitalia	4(1.1)	0.0–2.1
Blood in the urine, dysuria and itching of genitalia	10 (2.6)	1.1–4.2
Do not know	113(29.7)	25.2–34.7
**Is schistosomiasis cured**		
Yes	336 (88.4)	85.1–91.7
No	5(1.3)	0.3–2.4
Do not know	39(10.3)	7.3–13.4
**Ways to treat urogenital schistosomiasis**		
By swallowing tablets	303 (90.2)	86.8–93.4
By injection	16 (4.8)	2.6–7.2
By traditional medicine	3 (0.9)	0.0–2.1
Do not know	14 (4.2)	2.0–6.6
**Is schistosomiasis preventable**		
Yes	309(81.3)	77.5–85
No	17 (4.5)	2.5–6.6
Do not know	54(14.2)	10.8-18s
**Ways to prevent urogenital schistosomiasis**		
Treatment using anti-schistosomal medicine	85 (27.5)	22.3–32.4
By avoiding contact with unprotected water bodies	36(11.7)	8.2–15.9
Use of pipe water	3(1.0)	0.0–2.3
Use of latrines	10 (3.2)	1.6–5.6
By improving of personal hygiene	2(0.6)	0.0–1.9
All of the mentioned preventive measures	61 (19.7)	15.3–24.6
Treatment using anti-schistosomal medicine, by avoiding contact with unprotected water bodies and by improving of personal hygiene	42(13.6)	9.7–17.3
Treatment using anti-schistosomal medicine and use of clean water	31(10)	6.7–13.0
Treatment using anti-schistosomal medicine and by avoiding contact with unprotected water bodies	30(9.7)	6.4–13.0
Treatment using anti-schistosomal medicine, avoiding contact with unprotected water, use of clean water and latrines	4(1.3)	0.3–2.7
Do not know	5 (1.6)	0.3–3.3

### Influence of socio-demographic characteristics of the parents/guardians on the level of knowledge on urogenital schistosomiasis among the study participants

The low level of knowledge was higher among the parents/guardians aged 12–17 years (40%), who had never attended school (41.8%), and had been residents of Mtama district for < 11 years (52.7%). There was a statistically significant association between the level of knowledge and parents/guardians’ education level (p < 0.000), marital status (p = 0.026), the wards of the residency (p < 0.000), and years of residency in the Mtama District (p = 0.004) (Table 4).

**Table 4:** Influence of socio-demographic characteristics of the parents/guardians on the level of knowledge on the urogenital schistosomiasis among the study participants (n = 385).

Variable	Total	Low level of knowledge	Moderate level of knowledge	High level of knowledge	p-value
**Sex**					
Males	30	6(20)	21(70)	3(10)	0.506
Females	355	103(29)	211(59.4)	41(11.5)	
**Age (years)**					
12–17 (Teen)	10	4(40)	5(50)	1(10)	0.440
18–35 (Young adults)	266	70(26.3)	170(63.9)	26(9.8)	
36–55 (Middle-aged adults)	99	32(32.3)	52(52.5)	15(15.2)	
>56 (Older)	10	3(30)	5(50)	2(20)	
**Education level**					
Never attended school	110	46(41.8)	59(53.6)	5(4.5)	0.000*
Primary school	236	62(26.3)	146(61.9)	28(11.9)	
Secondary school	35	1(2.9)	25(71.4)	9(25.7)	
Post-secondary training	1	0(0.0)	0(0.0)	1(100)	
University	3	0(0.0)	2(66.7)	1(33.3)	
**Marital status**					
Married	257	78(30.4)	158(61.5)	21(8.2)	0.026*
Single	99	25(25.3)	59(59.6)	15(15.2)	
Divorced	24	5(20.8)	11(45.8)	8(33.3)	
Cohabiting	2	0(0.0)	2(100)	0(0.0)	
Widow(er)	3	1(33.3)	2(66.7)	0(0.0)	
**Occupation**					
Housewife	23	9(39.1)	12(52.2)	2(8.7)	0.201
Peasant	324	95(29.3)	194(59.9)	35(10.8)	
Petty business	32	5(15.6)	22(68.8)	5(15.6)	
Employed	6	0(0.0)	4(66.7)	2(33.3)	
**Residency (years)**					
≤ 11	55	29(52.7)	22(40)	4(7.3)	0.004*
12–17	17	5(29.4)	11(64.7)	1(5.9)	
18–35	234	54(23.1)	153(65.4)	27(11.5)	
36–55	71	19(26.8)	42(59.2)	10(14.1)	
> 56	8	2(25)	4(50)	2(25)	
**Wards of the residency**					
Longa	100	13(13)	60(60)	27(27)	0.000*
Nyengedi	165	37(22.4)	115(69.7)	13(7.9)	
Nyangamara	120	59(49.2)	57(47.5)	4(3.3)	

*Statistically significant (p < 0.05)

Of the total participants, 109 (28.3%, 95% CI 24.2–32.5) had a low knowledge, 232 (60.3%, 95% CI 55.8–65.2) had moderate knowledge, and 44 (11.4%, 95% CI 8.3–14.6) had high knowledge.

### Attitudes towards urogenital schistosomiasis among the parents/guardians of the under-fives

**Fig 2:**
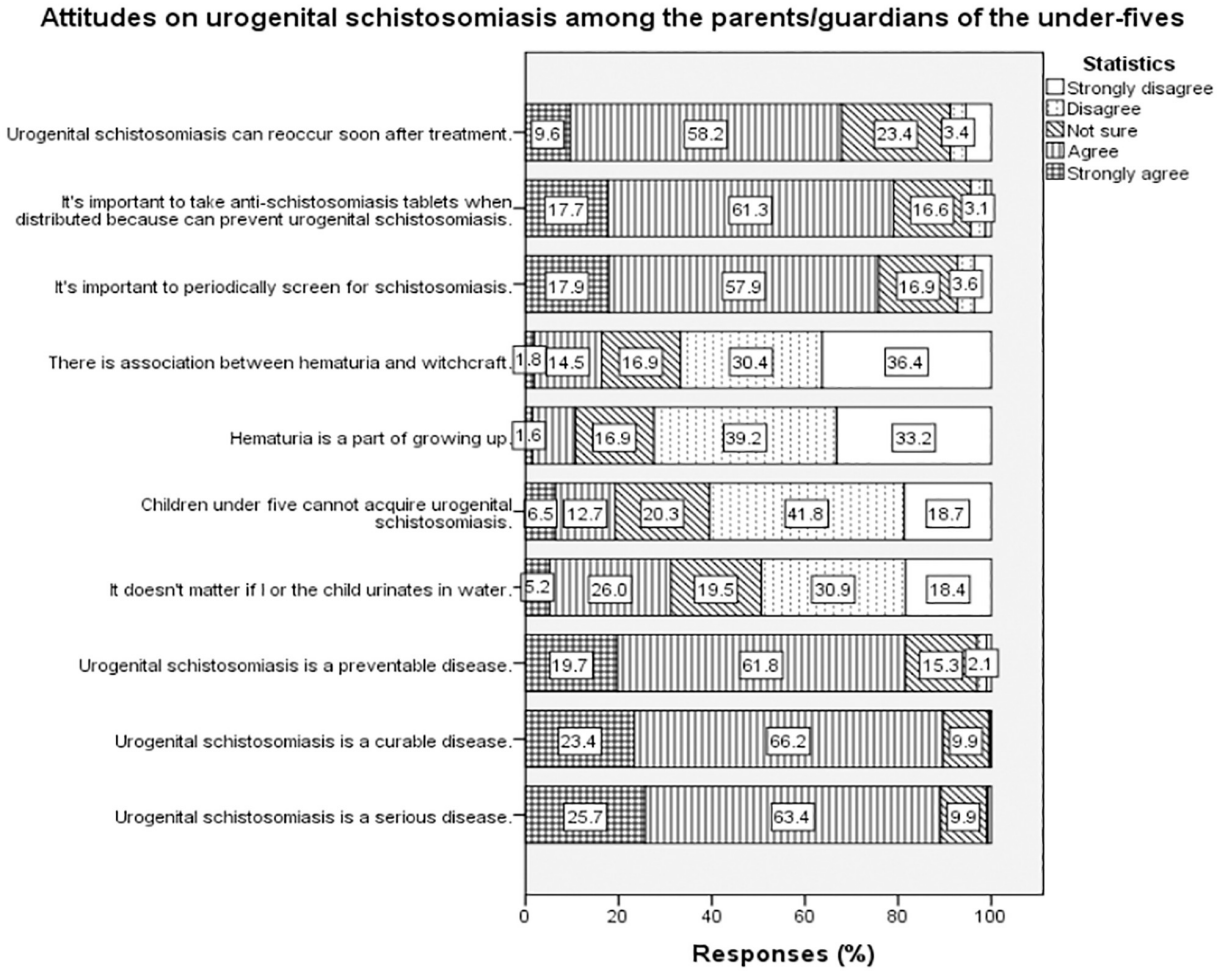
Attitudes on urogenital schistosomiasis among the parents/guardians of the under-fives.

Two-thirds (66.2%) and nearly two-thirds (61.8%) of the parents/guardians agreed urogenital schistosomiasis is a curable and preventable disease, respectively. More than half of the parents/guardians agreed to the importance of periodically screening for schistosomiasis (57.9%), and that the disease can reoccur soon after treatment (58.2%). Also, more than half the parents/ guardians (61.3%) agreed that it was crucial to take praziquantel when the drug was distributed for urogenital schistosomiasis prevention (Fig 2).

### Practices on urogenital schistosomiasis among the parents/guardians of the under-fives

**Fig 3:**
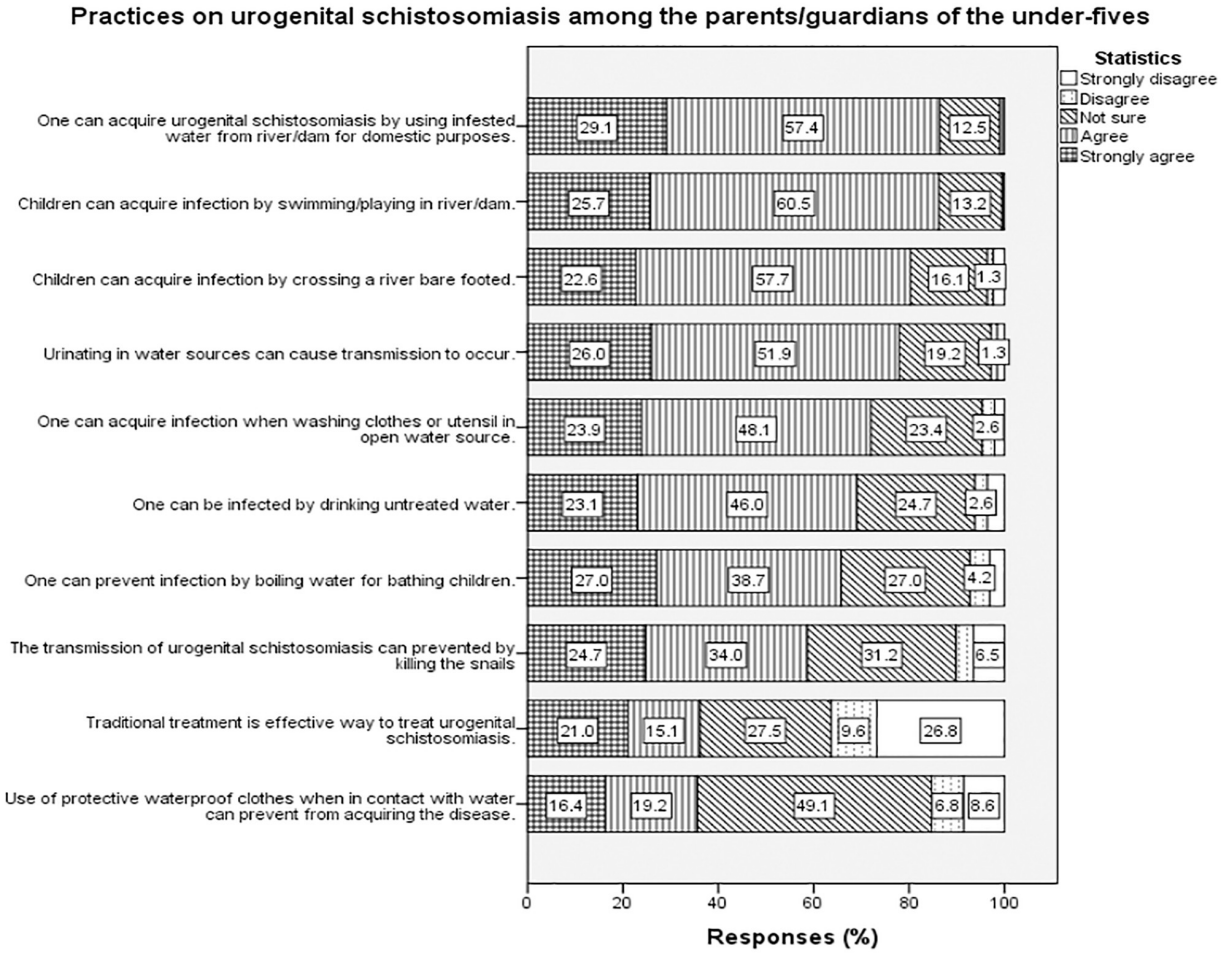
Practices on urogenital schistosomiasis among the parents/guardians of the under-fives.

More than half of the parents/guardians agreed the practice of urinating in water sources can perpetuate the transmission of urogenital schistosomiasis (51.9%), and children acquire the infection through swimming/playing in the water sources (60.5%). In terms of prevention, about one-third of the parents/guardians (34.3%) agreed that snail control would prevent urogenital schistosomiasis transmission, and the use of protective waterproof clothes and gumboots when in contact with water, could prevent the acquisition of the disease (49.1%) (Fig 3).

### Influence of socio-demographic characteristics on the attitudes and practices on urogenital schistosomiasis among the study participants

The age group, > 56 years, had higher participants with positive attitudes (70%) and appropriate practices (100%) for urogenital schistosomiasis compared to the remaining age groups. Also, the group of parents/guardians who never attended school had a high number of participants with negative attitudes (47.3%) and inappropriate practices (44.5%) toward urogenital schistosomiasis, compared to other education levels. There was a statistically significant association between the level of education of the parents/guardians and their attitudes (p = 0.029) and practices (p = 0.042) towards urogenital schistosomiasis (Table 5). Of the 385 parents/ guardians who participated in this study, 156 (40.5%) had negative attitudes towards urogenital schistosomiasis, while 229 (59.5%) had positive attitudes. In addition, a total of 132 (34.3%) parents/guardians had inappropriate practices towards urogenital schistosomiasis, and the remaining 253 (65.7%) had appropriate practices.

**Table 5:** Influence of socio-demographic characteristics of the parents/guardians on the attitudes and practices on the urogenital schistosomiasis among the study participants (n = 385).

Variable	Total	Negative attitude	Positive attitude	p-value	Inappropriate practice	Appropriate practice	p-value
**Sex**							
Males	30	9(30)	21(70)	0.152	5(16.7)	25(83.3)	0.034*
Females	355	147(41.4)	208(58.6)		127(35.8)	228(64.2)	
**Age (years)**							
12–17 (Teen)	10	5(50)	5(50)	0.423	2(20.0)	8(80.0)	0.089
18–35 (Young adults)	266	102(38.3)	164(61.7)		96(36.1)	170(63.9)	
36–55 (Middle- aged adults)	99	46(46.5)	53(53.5)		34(34.3)	65(65.7)	
> 56 (Older)	10	3(30)	7(70)		0(0.0)	10(100.0)	
**Education level**							
Never attended school	110	52(47.3)	58(52.7)	0.029*	49(44.5)	61(55.5)	0.042*
Primary school	236	97(41.1)	139(58.9)		75(31.8)	161(68.2)	
Secondary school	35	6(17.1)	29(82.9)		8(22.9)	27(77.1)	
Post-secondary training	1	0(0.0)	1(100.0)		0(0.0)	1(100)	
University	3	1(33.3)	2(66.7)		0(0.0)	3(100)	
**Marital status**							
Married	257	102(39.7)	155(60.3)	0.252	92(35.8)	165(64.2)	
Single	99	41(41.4)	58(58.6)		30(30.3)	69(69.7)	
Divorced	24	13(54.2)	11(45.8)		8(33.3)	16(66.7)	
Cohabiting	2	0(0.0)	2(100.0)		2(100)	0(0.0)	
Widow(er)	3	0(0.0)	3(100.0)		0(0.0)	3(100)	
**Occupation**							
Housewife	23	11(47.8)	12(52.2)	0.042*	11(47.8)	12(52.2)	0.034*
Peasant	324	137(42.3)	187(57.7)		115(35.5)	209(64.5)	
Petty business	32	8(25)	24(75)		6(18.8)	26(81.2)	
Employed	6	0(0.0)	6(100.0)		0(0.0)	6(100.0)	
**Residency (years)**							
≤ 11	55	25(45.5)	30(54.5)	0.772	32(58.2)	23(41.8)	0.000*
12–17	17	8(47.1)	9(52.9)		5(29.4)	12(70.6)	
18–35	234	92(39.3)	142(60.7)		77(32.9)	157(67.1)	
36–55	71	29(40.8)	42(59.2)		18(25.4)	53(74.6)	
> 56	8	2(25)	6(75)		0(0.0)	8(100.0)	
**Wards of the residency**							
Longa	100	27(27)	73(73)	0.002*	26(26.0)	74(74.0)	0.008*
Nyengedi	165	69(41.8)	96(58.2)		52(31.5)	113(68.5)	
Nyangamara	120	60(50)	60(50.0)		54(45.0)	66(55.0)	

*Statistically significant (p < 0.05)

### Water, sanitation, and hygiene status of the parents/guardians of the under-fives

The majority of the parents/guardians (80.5%) reported visiting water bodies and carrying their children to water bodies (75.2%), with rivers (73.9%) being the most frequently visited. In addition, nearly two-thirds of the parents/ guardians (62.6%) reported that their children urinated in water sources when playing due to the absence of latrines around the water sources (100%). A high prevalence of urogenital schistosomiasis was among the under-fives who visited water bodies with their parents (18.7%), played in the water (17.8%), and also urinated in the water (19.1%). There was a statistically significant association between visiting water body, source of water used at home (p < 0.000), and boiling water for bathing the child (p < 0.000) with the prevalence of urogenital schistosomiasis (Table 6).

**Table 6:** Water, sanitation and hygiene status of the parents/guardians of the under-fives (n = 385).

Variable	n (%)	*S*. *haematobium* positive	p-value
**Visit water body**			
Yes	310(80.5)	58(18.7)	0.044*
No	75(19.5)	7(9.3)	
**Waterbody visited**			
Dam	21(6.8)	2(9.5)	0.270
Pond water	29(9.4)	6(20.7)	
Irrigation scheme	4(1.3)	2(50)	
River	229(73.9)	43(18.8)	
Spring	8(2.6)	2(25)	
Dug well	10(3.2)	0(0.0)	
River, irrigation scheme, and pond water	9(2.9)	3(33.3)	
**Source of water at home**			
Tap	279(72.5)	34(12.2)	0.000*
Dug well	31(8.1)	7(22.6)	
Spring	5(1.3)	1(20.0)	
River	70(18.2)	23(32.9)	
**Carries the child when going to water sources**			
Yes	233(75.2)	46(19.7)	0.717
No	70(22.6)	11(15.7)	
Sometimes	7(2.3)	1(14.3)	
**Children playing in water**			
Yes	264(68.6)	47(17.8)	0.477
No	121(31.4)	18(14.9)	
**Children urinating in water when playing**			
Yes	241(62.6)	46(19.1)	0.233
No	118(30.6)	17(14.4)	
Sometimes	26(6.8)	2(7.7)	
**Children wearing shoes when crossing the water sources**			
Yes	192(49.9)	35(18.2)	0.482
No	193(50.1)	30(15.5)	
**Presence of latrines in water sources**			
Yes	0(0.0)	-	-
No	310(100.0)	58(18.7)	
**Place of urination when at water sources**			
In a nearby bush	257(82.9)	46(17.9)	0.273
In the water source	36(11.6)	10(27.8)	
I go back home	17(5.5)	2(11.8)	
**Use detergents when doing activities in water bodies**			
Yes	276(89.0)	53(19.2)	0.738
No	10(3.2)	1(10.0)	
Sometimes	24(7.7)	4(16.7)	
**Source of water for bathing the child**			
River	85(22.1)	21(24.7)	0.212
Dam	22(5.7)	2(9.1)	
Irrigation scheme	4(1.0)	1(25.0)	
Protected well	21(5.5)	4(19.0)	
Tap	253(65.7)	37(14.6)	
**Boiling water for bathing the child**			
Yes	193(50.1)	27(14.0)	0.000*
No	70(18.2)	26(37.1)	
Sometimes	122(31.7)	12(9.8)	

*Statistically significant (p < 0.05)

### Factors associated with ongoing transmission of urogenital schistosomiasis among the under-fives

Based on univariate logistic regression, ward of residency, level of parental/guardians’ knowledge, attitudes of the parents/guardians, the behavior of visiting the water bodies, type of water bodies’ visited and practice of boiling water for bathing the child were all statistically significantly associated with urogenital schistosomiasis infection (p < 0.005). After adjusting for the confounders, the behavior of visiting the water bodies and the type of the water body visited were statistically significantly associated with the ongoing transmission of urogenital schistosomiasis among the under-fives. Children of parents/guardians who practiced visiting water bodies with their children were more likely to have urogenital schistosomiasis than children of parents/guardians that do not practice this behavior (AOR: 1.44, 95% CI: 1.13–1.74). Also, the under-fives who visited the river with their parents/guardians were six times at higher risk of acquiring urogenital schistosomiasis (AOR: 6.00, 95% CI: 1.02–35.12) compared to the under-fives who visited other types of water bodies (Table 7).

**Table 7:** Univariate and multivariate regression analysis of the factors associated with ongoing transmission of urogenital schistosomiasis among the under-fives (n = 385).

Variable	Univariate analysis		Multivariate analysis	
	^a^COR (95% CI)	p-value	^b^AOR (95% CI)	p-value
**Sex of the children**				
Males	1(Ref)	0.449		
Females	0.81(0.48–1.39)			
**Age (months) of the children**				
12–35 (Young children)	1(Ref)	0.764		
36–59 (Pre-schoolers)	1.10(1.35–1.91)			
**Sex of the parents/guardians**				
Males	1(Ref)	0.590		
Females	1.35(0.45–4.00)			
**Age (years) of the parents/guardians**				
12–17 (Teen)	1(Ref)			
18–35 (Young adults)	0.95(0.19–4.84)	0.951		
36–55 (Middle aged adults)	1.20(0.66–2.17)	0.552		
>56 (Older)				
**Education level of the parents/guardians**	1(Ref)			
Never attended school	0.80(0.27–2.32)	0.679		
Primary school	0.82(0.20–2.23)	0.693		
Secondary school	0.94(0.38–3.45)	0.714		
Post-secondary training				
**Marital status of the parents/guardians**				
Married	1(Ref)		1(Ref)	
Single	7.03(0.43–10.65)	0.172	12.18(0.20–17.29)	0.231
Divorced	3.30(0.20–11.90)	0.405	10.77(0.17–16.82)	0.261
Cohabiting	1.67(0.10–9.06)	0.729	7.17(0.10–14.92)	0.362
**Occupation of the parents/guardians**				
Housewife	1(Ref)		1(Ref)	
Peasant	2.22(0.52–9.51)	0.282	1.20(0.17–8.39)	0.851
Petty business	1.67(0.71–3.91)	0.240	1.30(0.32–5.31)	0.716
**Residency (years) of the parents/guardians**				
≤ 11	1(Ref)		1(Ref)	
12–17	1.49(0.60–3.68)	0.391	1.46(0.39–5.44)	0.578
18–35	0.95(0.27–3.30)	0.930	1.01(0.22–4.58)	0.992
36–55	1.60(0.83–3.10)	0.163	2.29(0.98–5.36)	0.046
**Wards of the residency**				
Longa	1(Ref)		1(Ref)	
Nyengedi	0.11(0.05–0.236)	0.000*	0.07(0.01–0.33)	0.001*
Nyangamara	0.95(0.39–2.296)	0.906	0.41(0.10–2.01)	0.270
**Level of knowledge**				
Low level	1(Ref)			
Moderate level	4.0(1.68–9.70)	0.002*	0.80(0.20–2.9)	0.687
High level	2.5(1.20–5.24)	0.013*	1.22(0.42–3.6)	0.711
**Classification of attitudes**				
Positive attitudes	1(Ref)		1(Ref)	
Negative attitudes	1.70(0.10–3.18)	0.042*	1.44(0.65–3.18)	0.365
**Classification of practices**				
Appropriate practices	1(Ref)		1(Ref)	
Inappropriate practices	1.57(0.86–2.90)	0.138	1.50(0.63–3.61)	0.362
**Visit water bodies**				
Yes	1.49(1.20–1.50)	0.002*	1.44(1.13–1.74)	0.034*
No	1(Ref)		1(Ref)	
**Type of water body visited**				
River, irrigation scheme and pond	1(Ref)		1(Ref)	
Dam	0.98(0.19–5.10)	0.979	1.75(0.13–23.89)	0.675
Irrigation scheme	0.10(0.01–0.85)	0.035*	1.92(0.09–41.24)	0.678
River	0.45(0.19–1.30)	0.041*	6.00(1.20–35.12)	0.043*
Spring	0.31(0.10–1.83)	0.196	3.45(0.24–49.31)	0.362
Pond	0.40(0.12–0.85)	0.125	1.28(0.13–13.00)	0.835
**Carries the child when going to water sources**				
Yes	1(Ref)			
No	0.85(0.10–8.00)	0.888		
Sometimes	1.90(0.10–10.84)	0.942		
**Children playing in water**				
Yes	1.1(0.45–2.9)	0.785		
No	1(Ref)			
**Children urinating in water when playing**				
Yes	1(Ref)			
No	0.42(0.10–1.90)	0.261		
Sometimes	0.57(0.11–2.80)	0.487		
**Place of urination when at water bodies**				
I go back home	1(Ref)		1(Ref)	
In a nearby bush	0.65(0.17–3.00)	0.585	0.62(0.06–6.55)	0.693
In the water source	0.35(0.10–1.82)	0.211	0.53(0.04–6.33)	0.615
**Source of water for bathing the child**				
Tap	1(Ref)			
River	0.76(0.39–1.47)	0.407		
Dam	2.00(0.43–9.24)	0.379		
Irrigation scheme	0.80(0.10–8.67)	0.856		
Protected well	2.04(0.42–9.94)	0.376		
**Boiling water for bathing the child**				
Sometimes	1(Ref)		1(Ref)	
Yes	0.63(0.28–1.43)	0.268	0.70(0.26–1.82)	0.460
No	0.18(0.10–0.45)	0.000*	0.29(0.10–0.79)	0.001

^*a*^*COR* Stands for Crude Odds Ratios,

^*b*^*AOR* Stands for Adjusted Odds Ratios,

*Statistically significant (p < 0.05)

## Discussion

This study presents baseline epidemiological data for urogenital schistosomiasis in under-fives in the Mtama district in the Lindi region of Tanzania. The data shows that 16.9% of the under-fives were infected with S. *haematobium* and about a quarter of those infected had heavy infections. The revealed infection prevalence is higher than the prevalence reported in studies conducted in endemic areas of Tanzania, Malawi, and Zimbabwe [14,18,24]. The prevalence and intensity of urogenital schistosomiasis were higher in preschoolers (36–59 months) compared to young children (12–35 months) but the differences were not statistically significant. Evidence has it infections can be acquired at an early age (within the first year of a child’s life) in endemic areas, and intensity increases as the child grow [6,7]. The under-five males had higher infection intensity compared to females; this is consistent with a systematic review that reported that males were significantly more likely to be infected with *S*. *haematobium* compared to females [25]. The under-fives are at high risk of acquiring urogenital schistosomiasis when going to water sources with their parents/guardians or when bathed with infested water [5]. This scenario was similar to the situation in the Mtama district. Also, in the Mtama district, the children aged 36–59 months had a practice of visiting the water sources with their brothers/sisters (school-aged children) to play and learn how to swim. Hence, at higher risk of being exposed to *S*. *haematobium*. This shows urogenital schistosomiasis is a significant public health problem in the under-fives and stresses the need for the under-fives inclusion in the ongoing schistosomiasis control program. There was a statistically significant variation in the prevalence of *S*. *haematobium* between the selected wards, with the highest prevalence at the Longa ward (43%) and the lowest at the Nyangamara ward (7.5%). The presence of several water bodies in the Longa ward compared to the rest and the proximity of the villages in the Longa ward to the water bodies could be the reason for the observed variation.

The vast majority (98.7%) of the parents/guardians were aware of urogenital schistosomiasis. This is because the disease has been endemic in the Mtama district for more than three decades with more than one decade of ongoing schistosomiasis control programmes with praziquantel treatment for school-aged children [15]. Despite high awareness about urogenital schistosomiasis among the parents/guardians, more than a quarter of them still had a low level of knowledge regarding the disease. About 40.5% had negative attitudes, and more than one-third had inappropriate practices (34.3%) regarding the prevention and control of the disease. The findings are consistent with other studies conducted in Tanzania, Cameroon, and Zimbabwe [5,26,27]. Low level of knowledge (40%) and negative attitudes (50%) were high among the teens aged 12–17 years, which probably was contributed by the lack of awareness of the disease. The low knowledge and negative attitudes among the teens could negatively impact the practices towards disease prevention. The only teenager with high knowledge was aged 15 years who had secondary education. In the urogenital schistosomiasis endemic areas, the lower level of knowledge increased negative attitudes and misconceptions, leading to inappropriate prevention practices [28]. Also, supported by the findings from Tanzania and Cameroon [26–28]. The observed low level of knowledge coupled with negative attitudes and inappropriate practices in the Mtama district is due to the lack of comprehensive health education on urogenital schistosomiasis. Therefore, there is a need to initiate social and behavior change communication programs to supplement parents/guardians’ knowledge on urogenital schistosomiasis to appropriately change the negative attitudes on the disease to a level that influences appropriate practices to prevent the disease. Social and behavior change communication programs should focus on improving the knowledge of disease transmission and prevention. Also, health education should address the misconceptions on the local beliefs about urogenital schistosomiasis, the role of *Bulinus* snail species in transmission, screening, and recurrence of the disease.

*S*. *haematobium* is more prevalent in areas with inadequate water supplies, poor sanitation, and hygiene [29]. The scarcity of clean water in the Mtama district has resulted in some community members depending on unprotected water sources [17]. The majority of the parents/guardians (80.5%) still visited the water bodies despite having access to tap water (72.5%) as a source of water at home. However, there was a lack of taps in most of the households, causing some of the community members to buy tap water from water vendors. Hence, to minimize water bills, tap water is reserved for cooking, drinking, and sometimes bathing the children. The remaining domestic chores are being done in unprotected water sources hence increasing the risk for transmission of urogenital schistosomiasis. The prevalence of urogenital schistosomiasis was statistically higher among the under-fives accompanying their parents/guardians to water sources (19.7%); this is because, at the water sources, the children play in the shallow sides of the water thus, are exposed to the infested water. The finding is similar to the observation in Zimbabwe [5,8]. The absence of latrines at the water sources facilitates the practice of open urination and defecation, causing contamination of the water sources in the Mtama district. Adequate sanitation is crucial in the control of urogenital schistosomiasis aiming at the prevention of water sources contamination, and it was stated that the presence of adequate sanitation significantly lowers the odds of *S*. *haematobium* infection [30]. The higher prevalence was observed in under-fives having unhygienic behavior of urinating in the water (19.1%) during bathing, swimming, and crossing the water sources barefooted (18.2%). These unhygienic behavior have been reported to perpetuate the transmission and acquisition of the *S*. *haematobium* [2,5,30].

The analysis of the risk factors revealed a significant association between the prevalence of *S*. *haematobium* and factors such as wards of residency, level of knowledge and attitudes of the parents/guardians, the practice of visiting the water bodies, type of the water bodies visited, and boiling water for bathing children. This conforms to the studies conducted in sub-Saharan Africa [2,8,30–32]. In multivariate logistic regression, the odds of *S*. *haematobium* infection were high in under-fives whose parents/guardians had the practice of visiting the water bodies (AOR = 1.44), rivers particularly (AOR = 6.00), while the odds of the infection were low in children bathed with boiled water (AOR = 0.29). The higher odds of the infection were due to the practice of the parents/guardians visiting the water bodies with their under-fives [5]. The lower odds of infection in under-fives bathed with boiled water was due to the water treatment which made water free of cercariae and hence safe for domestic use. However, this method should not substitute the use of chemotherapy, intermediate host control, and WaSH [33].

Although informative, this current study was limited by the actual sample size of the under-five and their parents/guardians recruited in this study. This was lower than the estimated sample size for the validity and generalizability of the findings. Also, it was challenging to ensure that the urine collected belonged to the sampled under-five. The collection of single urine samples and the lack of more sensitive diagnostic tools may have underestimated the burden of urogenital schistosomiasis in the study area. However, the data provides a holistic picture of the epidemiology of urogenital schistosomiasis among the under-fives in the Mtama district. Some of the responses in the WaSH section could be biased and unreliable compared to the observation using the prepared checklist.

## Conclusions and recommendations

This study has revealed ongoing transmission of urogenital schistosomiasis among under-fives in the Mtama district. The risk factors for the transmission of urogenital schistosomiasis in the study area were inadequate knowledge of the parents/guardians about the disease, negative attitudes and inappropriate preventive practices, intense human water contact, inadequate supply of affordable clean water, inadequate sanitation at the water bodies, and unhygienic practices. Therefore, the government, through the Neglected Tropical Diseases Control Program, should do regular screening for urogenital schistosomiasis in the under-fives followed by appropriate treatment. The Lindi Urban Water Supply and Sanitation Authority should supply adequate tap water and construct latrines at the water bodies to stop the unhygienic practices contaminating the water bodies and leading to ongoing transmission. The government, in collaboration with non-governmental organizations, should initiate schistosomiasis health education campaigns aiming to provide health education to the parents/guardians of the under-fives on all aspects of the disease from the transmission to prevention. Health education should not aim to improve knowledge only but also to change negative attitudes, misconceptions on transmission, and inappropriate practices that could lead to the acquisition of the disease by the under-fives.

## Supporting information

S1 File Urine analysis form. (DOCX)

S2 File Mothers/Caregivers questionnaire (English). (DOCX)

S3 File Mothers/Caregivers questionnaire (Kiswahili). (DOCX)

S4 File The quality control results for the urine filtration technique. (XLSX)

S1 Data The data set used for analysis. (XLSX)

S1 Table The classification of infection intensity according to socio-demographic characteristics of the under-fivess. (DOCX)
