# Supplementary material for: Prevalence and risk factors of urogenital schistosomiasis among under-fives in Mtama District in the Lindi region of Tanzania
Source: PLoS Negl Trop Dis. 2022 Apr 20;16(4):e0010381. doi: 10.1371/journal.pntd.0010381 (PMC9060350; doi:10.1371/journal.pntd.0010381)
Supplement: S1 File — (DOCX) [file pntd.0010381.s001.docx]

# S1_File: Urine analysis form

ID Number……………….. Ward……………….……………

Village……………………. Date…………/……./…………... Sex………………………… Age (month)…………………….

| **S/N** | **Variable to be examined** | **Result** |
| --- | --- | --- |
| 1 | Macroscopic examination of urine   - Color - Presence of blood |  |
| 2 | Microhaematuria examination |  |
| 3 | Microscopic examination of *S. haematobium* ova   - Number of eggs/10 mls of urine - Categorization of Intensity |  |

Name of the investigator……………………………… Signature………………………..
