# Supplementary material for: Prevalence and risk factors of urogenital schistosomiasis among under-fives in Mtama District in the Lindi region of Tanzania
Source: PLoS Negl Trop Dis. 2022 Apr 20;16(4):e0010381. doi: 10.1371/journal.pntd.0010381 (PMC9060350; doi:10.1371/journal.pntd.0010381)
Supplement: S2 File — (DOCX) [file pntd.0010381.s002.docx]

# S2_File: MOTHERS/CAREGIVERS QUESTIONNAIRE (English version)

Questionnaire ID no…………………………

Name of Interviewer………………………..

Date of Interview……………………………..

**PART A**

**Demographic information**

| 1 | Ward Name |  |
| --- | --- | --- |
| 2 | Village Name |  |
| 3 | Sex | Male…………………1  Female……………….2 |
| 4 | Age (years) |  |
| 5 | Marital status | Married………………1  Single…………….......2  Divorced……………..3  Cohabiting……….......4  Widow(er)…..………..5 |
| 6 | Education level | Never attended school………..1  Primary ………………............2  Secondary…………………….3  Post-secondary training…… ...4  University…………………….5 |
| 7 | Occupation | Housewife………………..…..1  Peasant……………………….2  Petty business………………..3  Fishing……………………….4  Employed……………………5  Other (specify)………......…...6 |
| 8 | Duration lived in the village |  |

**PART B**

**Knowledge of Urogenital Schistosomiasis**

1. Have you ever heard of the disease called urogenital schistosomiasis?
2. Yes…………….1
3. No……………..2
4. If yes, where did you get the information about urinary schistosomiasis?
5. Dispensary…... …………………………1
6. Mass media……………………………...2
7. Community health workers……………...3
8. Friend……………………………………4
9. Others (specify)……………………….....5
10. Have you have ever suffered from urinary schistosomiasis?
11. Yes…………………………………1
12. No………………………………….2
13. Do not remember ………………….3
14. What is the causative agent of urogenital schistosomiasis?
15. Bacteria…... …………….1
16. Virus…………………......2
17. Parasite...………………...3
18. Fungi…………………….4
19. Do not know……………..5
20. How is urogenital schistosomiasis transmitted?
21. Through eating contaminated foodstuffs…………1
22. Through sexual intercourse……………………….2
23. Contacting infected water………………………...3
24. Drinking dirty water………………………………4
25. Do not know………………………………………5
26. Snails play part in transmission of urogenital schistosomiasis
27. Yes………………………..1
28. No………………………...2
29. Do not know………………3
30. Which symptoms do you think are related to urogenital schistosomiasis?
31. Urine in the blood…………………………………1
32. Stomach ache……………………………………...2
33. Dysuria…………………………………………….3
34. Diarrhea …………………………………………...4
35. Coughing…………………………………………..5
36. Headache…………………………………………..6
37. Itching……………………………………………...7
38. Fever…………...…………………………………...8
39. Blood in the feces……………...…………………...9
40. Do not know…………………………………….....10
41. Can someone with urinary schistosomiasis be cured?
42. Yes…………………………………1
43. No………………………………….2
44. Do not know …………………...….3

1. How can it be treated?
2. By swallowing tablets ……………………………..1
3. By injection………………………………………...2
4. By being operated …………………………………3
5. By traditional medicine…………………………….4
6. Do not know………………………………………..5
7. Is urogenital schistosomiasis a preventable disease?
8. Yes………………………………1
9. No…………………………….....2
10. Do not know……………………..3
11. How can you prevent and control schistosomiasis?
12. Treatment using anti-schistosomal medicine……………………...………..1
13. By avoiding contact with unprotected water bodies…………………..……2
14. Use of pipe water……………………………………………………….…..3
15. Use of latrines ……………………………………………………………...4
16. By improving of personal hygiene …………..……………………………..5
17. Do not know………………………………………………………………...6

**PART C**

**WASH practices associated with transmission of urogenital schistosomiasis**

1. What is the main source of water used by members of your household for purposes such as cooking, hand washing, cleaning the house etc?
2. Piped water………………………………………………..1
3. Dug well ………………………………………………….2
4. Water from spring ………………………………………..3
5. Water kiosk ………………………………………………4
6. Open water sources …………………….…………………5 (river, stream, dam, irrigation scheme, ponds etc).
7. Others, specify……………………………………………6
8. Do you visit any water body or participate in any economic activity that pre disposes you into contact with water?
9. Yes……………………………………..1
10. No……………………………………...2
11. If yes which water bodies do you come into contact frequently?
12. River …………………………………………1
13. Irrigation scheme …………………………….2
14. Pond water……………………………………3
15. Dam….………………………………………..4
16. Spring.......…………………………………….5
17. Others (specify)……………..…………….…..6
18. Do you carry your child with you to water sources?
19. Yes……………………………………1
20. No …………………………………….2
21. Sometimes……………………..……...3
22. Do children go and play in the surface water present nearby the village?
23. Yes ………………………………………………1
24. No………………………………………………..2
25. Do children wear shoes while walking along the shores of water surfaces?
26. Yes ………………………………………………1
27. No………………………………………………..2
28. Is there any latrine around the areas you conduct these activities?
29. Bathing i) Yes……………..1 (ii) No…………….2
30. Fetching water i) Yes……………..1 (ii) No…………….2
31. Farming i) Yes…………….1 (ii) No…………….2
32. Fishing i) Yes…………….1 (ii) No…………….2
33. Swimming i) Yes…………….1 (ii) No…………….2
34. Washing clothes i) Yes…………….1 (ii) No…………….2
35. If there are no toilets, where do you urinate during the above activities?
36. In the nearby bush ………………………………..1
37. Around the water source …………………………2
38. I go back home …………………………………...3
39. Do children urinate in water while playing/bathing?
40. Yes………………………………..1
41. No………………………………...2
42. Sometimes………………………...3
43. Do you use detergents or soaps during domestic chores in water sources?
44. Yes………………………………..1
45. No………………………………...2
46. Sometimes………………………...3
47. What is the usual source of water for bathing the child?
48. River…………………………………...1
49. Dam……………………………………2
50. Irrigation schemes……………………...3
51. Protected well………………………….4
52. Tap water………………….…………...5
53. Do you boil water for bathing the child?
54. Yes………………………………..1
55. No………………………………...2
56. Sometimes………………………..3

**PART D**

**Attitudes on urogenital schistosomiasis [Am going to read several statements regarding urogenital schistosomiasis please rate the degree which you are agree or disagree with each]**

| **CODE** | **QUESTION** | **Strongly**  **Disagree** | **Disagree** | **Not sure** | **Agree** | **Strongly**  **Agree** |
| --- | --- | --- | --- | --- | --- | --- |
| 1 | Urogenital schistosomiasis is a serious disease. | **1** | **2** | **3** | **4** | **5** |
| 2 | Urogenital schistosomiasis is a curable disease. | **1** | **2** | **3** | **4** | **5** |
| 3 | Urogenital schistosomiasis is a preventable disease. | **1** | **2** | **3** | **4** | **5** |
| 4 | It doesn't matter if I or the child urinates in water. | **1** | **2** | **3** | **4** | **5** |
| 5 | Children under five cannot acquire urogenital schistosomiasis. | **1** | **2** | **3** | **4** | **5** |
| 6 | Hematuria is a part of growing up. | **1** | **2** | **3** | **4** | **5** |
| 7 | There is association between hematuria and witchcraft. | **1** | **2** | **3** | **4** | **5** |
| 8 | It's important to periodically screen for schistosomiasis. | **1** | **2** | **3** | **4** | **5** |
| 9 | It's important to take anti-schistosomiasis tablets when distributed because can prevent urogenital schistosomiasis. | **1** | **2** | **3** | **4** | **5** |
| 10 | Urogenital schistosomiasis can reoccur soon after treatment. | **1** | **2** | **3** | **4** | **5** |

**PART E**

**Practices towards urogenital schistosomiasis [Am going to read several statements regarding urogenital schistosomiasis please rate the degree which you are agree or disagree with each]**

| **CODE** | **QUESTION** | **Strongly**  **Disagree** | **Disagree** | **Not sure** | **Agree** | **Strongly**  **Agree** |
| --- | --- | --- | --- | --- | --- | --- |
| 1 | One can acquire urogenital schistosomiasis by using infested water from river/dam for domestic purposes. | **1** | **2** | **3** | **4** | **5** |
| 2 | Children can acquire infection by swimming/playing in river/dam. | **1** | **2** | **3** | **4** | **5** |
| 3 | Children can acquire infection by crossing a river bare footed. | **1** | **2** | **3** | **4** | **5** |
| 4 | Urinating in water sources can cause transmission to occur. | **1** | **2** | **3** | **4** | **5** |
| 5 | One can acquire infection when washing clothes or utensil in open water source. | **1** | **2** | **3** | **4** | **5** |
| 6 | One can be infected by drinking untreated water. | **1** | **2** | **3** | **4** | **5** |
| 7 | One can prevent infection by boiling water for bathing children. | **1** | **2** | **3** | **4** | **5** |
| 8 | The transmission of urogenital schistosomiasis can prevented by killing the snails | **1** | **2** | **3** | **4** | **5** |
| 9 | Traditional treatment is effective way to treat urogenital schistosomiasis. | **1** | **2** | **3** | **4** | **5** |
| 10 | Use of protective waterproof clothes when in contact with water can prevent from acquiring the disease. | **1** | **2** | **3** | **4** | **5** |
