# Supplementary material for: Prevalence and risk factors of urogenital schistosomiasis among under-fives in Mtama District in the Lindi region of Tanzania
Source: PLoS Negl Trop Dis. 2022 Apr 20;16(4):e0010381. doi: 10.1371/journal.pntd.0010381 (PMC9060350; doi:10.1371/journal.pntd.0010381)
Supplement: S3 File — (DOCX) [file pntd.0010381.s003.docx]

**S3_File: DODOSO LA WAMAMA/WALEZI WA WATOTO WALIO NA UMRI CHINI YA MIAKA MITANO (Kiswahili version)**

Namba ya utambulisho ya hojaji …………………………

Jina la mhojaji …………………………………………….

Tarehe ya mahojiano ……………………………………...

**SEHEMU A: Taarifa za Kidemografia**

| 1 | Jina la Kata |  |
| --- | --- | --- |
| 2 | Jina la kijiji |  |
| 3 | Jinsia | Mwanaume…………1  Mwanamke ...……….2 |
| 4 | Umri **[**Miaka] |  |
| 5 | Hali ya Ndoa | Ndoa……………………...1  Hujaoa/olewa..…………....2  Talaka……………..............3  Kimada…………………....4  Mjane/Mgane…………..….5 |
| 6 | Kiwango cha elimu | Hajawai kwenda shule …...1  Elimu ya msingi…… ……..2  Elimu ya sekondari……….3  Elimu ya ufundi…….…….4  Elimu ya juu….…….…......5 |
| 7 | Kazi unayofanya | Mama wa nyumbani ……..1  Mkulima……….………….2  Biashara ndogo ndogo……3  Uvuvi…………….………..4  Ameajiriwa…….………….5  Nyingine (taja)……………6 |
| 8 | Muda ulioishi katika kijiji hiki |  |

**SEHEMU B: UFAHAMU JUU YA UGONJWA WA KICHOCHO CHA MKOJO**

1. Je umewahi kusikia juu ya ugonjwa uitwao kichocho cha mkojo?
2. Ndiyo …………….1
3. Hapana…………...2
4. Kama ndio unaufahamu, je ulipata wapi taarifa kuhusiana na ugonjwa huu?
5. Zahanati…... ……………….1
6. Vyombo vya habari……..…..2
7. Afisa afya wa kijiji………….3
8. Rafiki……………….………4
9. Kwingine (taja)…………......5
10. Je umewahi kuugua kichocho cha mkojo?
11. Ndiyo…………………….…1
12. Hapana ………….………….2
13. Sikumbuki ………………….3
14. Je ni kimelea kipi kinasababisha ugonjwa wa kichocho cha mkojo?
15. Bakteria…... …………….1
16. Virusi………………….....2
17. Minyoo...…………………3
18. Kuvu (Fangasi)…………...4
19. Sijui……………………....5
20. Ni kwa njia ipi kichocho cha mkojo kinaambukizwa?
21. Kupitia kula vyakula vichafu……………………..1
22. Kujamiana………………...………………………2
23. Kugusa/ kukanyaga maji machafu …………….…3
24. Kunywa maji machafu …………………………...4
25. Sijui……………………………………………….5
26. Je, konokono wanahusika katika kusambaza vimelea vya kichocho cha mkojo?
27. Ndiyo.………………..1
28. Hapana……..………...2
29. Sijui…………………..3
30. Je ni dalili zipi unadhani zinahusiana na ugonjwa wa kichocho cha mkojo?
31. Damu kwenye mkojo………………………………1
32. Maumivu ya tumbo…………………..…………....2
33. Maumivu wakati wa kukojoa…………………..…..3
34. Kuharisha ……………………………………….....4
35. Kukohoa…………………………………………....5
36. Kuumwa kichwa…………………………………....6
37. Kuwashwa………………………………………….7
38. Homa…………...…………………..……………….8
39. Damu kwenye kinyesi……………………..………...9
40. Sijui…………………………………………….......10
41. Je mtu mwenye kichocho cha mkojo anaweza kutibiwa na kupona?
42. Ndiyo………………….…1
43. Hapana………..………….2
44. Sijui ………………….......3

1. Ni kwa njia gani kichocho cha mkojo kinaweza kutibiwa?
2. Kwa kumeza vidonge ……………….1
3. Kuchomwa sindano..………………..2
4. Kufanyiwa upasuaji ………………...3
5. Kwa kutumia dawa asilia……………4
6. Sijui…………………………………..5
7. Je kichocho cha mkojo kinazuilika?
8. Ndiyo….………………1
9. Hapana………………...2
10. Sijui……………………3
11. Ni kwa jinsi gani unaweza kuzuia na kudhibiti ugonjwa wa kichocho cha mkojo?
12. Kwa kutumia dawa ya kichocho……………………...…………………....1
13. Kuepuka kugusana/kutumia vyanzo vya maji visivyo salama ….…………2
14. Kutumia maji ya bomba ……………………………………………………3
15. Kutumia vyoo ………………………………………………………………4
16. Kuimarisha usafi binafsi …………………………………………….……..5
17. Sijui…………………………………………………………………………6

**SEHEMU C: Mahusiano kati ya maji, usafi wa mazingira na usafi binafsi unavyohusiana na maambukizi ya kichocho cha mkojo**

1. Ni chanzo kipi cha maji mnatumia kwa ajili ya shughuli mbalimbali za nyumbani kama kupika, kufanya usafi, kunawa mikono n.k?
2. Maji ya bomba…………………………………………..1
3. Kisima cha kuchimba…………………..……………….2
4. Maji kutoka kwenye chemchem ………………………..3
5. Maji kutoka kwenye kioski……..………………………4
6. Vyanzo vya maji vilivyo wazi …………………………5 (mto, vijito, bwawa, madimbwi, mfumo wa umwagiliaji etc).
7. Nyingine , (taja)……………………………………….....6
8. Je huwa unatembelea chanzo chochote cha maji au kushiriki kwenye shughuli zozote za kiuchumi zinazokufanya ukanyage maji?
9. Ndiyo………………………………………….1
10. Hapana………………………………………...2
11. Kama jibu lako ni ndiyo, je ni vyanzo vipi vya maji unavyotumia mara kwa mara?
12. Mto …………………………………………..1
13. Mfumo wa umwagiliaji………….…………...2
14. Madimbwi …………………………………...3
15. Bwawa….……………………………………4
16. Chemchemu.......…………………………..…5
17. Nyingine (taja)……………..………………...6
18. Je unaenda na mtoto wako kwenye vyanzo vya maji?
19. Ndiyo ………..…………………………...1
20. Hapana……………………………………2
21. Wakati mwingine…………………………3
22. Je watoto huwa wanaenda kuchezea kwenye vyanzo vya maji vilivyopo kijijini hapa?
23. Ndiyo………………………………………………1
24. Hapana …………………...………………………..2
25. Je watoto huwa wanavaa viatu wanpotembea kwenye kingo za vyanzo vya maji?
26. Ndiyo ………………………………………………1
27. Hapana……………………………………………...2
28. Je kuna choo kwenye maeneo unayofanyia shughuli hizi?
29. Kuoga i) Ndiyo…………….1 (ii) Hapana……………..2
30. Kuchota maji i) Ndiyo…………….1 (ii) Hapana……………..2
31. Kulima i) Ndiyo…………….1 (ii) Hapana……………..2
32. Kuvua samaki i) Ndiyo…………….1 (ii) Hapana……………..2
33. Kugelea i) Ndiyo…………….1 (ii) Hapana………… .....2
34. Kufua nguo i) Ndiyo…………….1 (ii) Hapana………...…...2
35. Je kama hakuna choo unakojoa wapi wakati unafanya shughuli hizo hapo juu?
36. Kwenye kichaka kilicho karibu ………………………………..1
37. Karibu na/ au kwenye chanzo cha maji ………………………..2
38. Narudi nyumbani ………………… ……………………….…..3
39. Je watoto huwa wanakojoa kwenye maji wakati wanacheza/wanaoga?
40. Ndiyo………………………………..1
41. Hapana……………………………....2
42. Wakati mwingine…………………….3
43. Je mnatumia sabuni unapofanya kazi zako za nyumbani kama kufua / kuosha vyombo kwenye vyanzo vya maji?
44. Ndiyo……………………………….1
45. Hapana……………………………...2
46. Wakati mwingine……………………3
47. Je ni chanzo kipi cha maji unatumia kumuogesha mtoto?
48. Mto …………………………….……….1
49. Bwawa………………………………….2
50. Mfumo wa umwagiliaji…………………3
51. Kisima kilichofunikwa………………….4
52. Maji ya bomba………………….……….5
53. Je unachemsha maji ya kumuogesha mtoto?
54. Ndiyo…………………..………………..1
55. Hapana…………………...……………...2
56. Wakati mwingine ………………….……3

**SEHEMU D: Maswali ya mwelekeo [Nitasoma sentensi kadhaa zinazohusiana na ugonjwa wa kichocho cha mkojo, tafadhali chagua kiwango unachokubaliana au kukataa kwa kila sentensi.]**

| **NAMBA** | **SWALI** | **Sikubaliani kabisa** | **Sikubaliani** | **Sinauwakika** | **Nakubaliana** | **Nakubaliana**  **Kabisa** |
| --- | --- | --- | --- | --- | --- | --- |
| 1 | Kichocho cha mkojo ni ugonjwa mbaya. | **1** | **2** | **3** | **4** | **5** |
| 2 | Kichocho cha mkojo ni ugonjwa unaotibika. | **1** | **2** | **3** | **4** | **5** |
| 3 | Kichocho cha mkojo kinazuilika. | **1** | **2** | **3** | **4** | **5** |
| 4 | Haijalishi kama mimi au mtoto akikojoa kwenye maji. | **1** | **2** | **3** | **4** | **5** |
| 5 | Watoto walio chini ya umri wa miaka mitano hawawezi kupata kichocho cha mkojo. | **1** | **2** | **3** | **4** | **5** |
| 6 | Kukojoa damu ni sehemu ya ukuaji. | **1** | **2** | **3** | **4** | **5** |
| 7 | Kuna mahusiano kati ya kukojoa damu na imani za kishirikina. | **1** | **2** | **3** | **4** | **5** |
| 8 | Ni muhimu kupima kichocho cha mkojo mara kwa mara. | **1** | **2** | **3** | **4** | **5** |
| 9 | Ni muhimu kumeza vidonge vya kuzuia maambukizi ya kichocho cha mkojo. | **1** | **2** | **3** | **4** | **5** |
| 10 | Maambukizi ya ugonjwa wa kichocho cha mkojo yanaweza kujitokeza tena mda mfupi baada ya matibabu. | **1** | **2** | **3** | **4** | **5** |

**SEHEMU E: Maswali ya mazoea/tabia [Nitasoma sentensi kadhaa zinazohusiana na ugonjwa wa kichocho cha mkojo, tafadhali chagua kiwango unachokubaliana au kukataa kwa kila sentensi.]**

| **NAMBA** | **SWALI** | **Sikubaliani kabisa** | **Sikubaliani** | **Sina uwakika** | **Nakubaliama** | **Nakubaliana kabisa** |
| --- | --- | --- | --- | --- | --- | --- |
| 1 | Mtu anaweza kupata kichocho kwa kutumia maji yenye vimelea vya ugonjwa kwenye mito/ mabwawa kwa shughuli mbalimbali za nyumbani. | **1** | **2** | **3** | **4** | **5** |
| 2 | Mtoto anaweza kupata kichocho cha mkojo kwa kuogelea au kucheza kwenye maji ya mto/bwawa | **1** | **2** | **3** | **4** | **5** |
| 3 | Mtoto anaweza kupata kichocho kwa kufuvuka mto miguu peku bila kuvaa viatu. | **1** | **2** | **3** | **4** | **5** |
| 4 | Kukojoa kwenye vyanzo vya maji kunaweza kusababisha maambukizi kutokea. | **1** | **2** | **3** | **4** | **5** |
| 5 | Mtu anaweza kupata kichocho cha mkojo anapokuwa bwawani/mtoni anafua nguo au kuosha vyombo. | **1** | **2** | **3** | **4** | **5** |
| 6 | Mtu anaweza kupata ugonjwa kwa kunywa maji yasio safi na salama. | **1** | **2** | **3** | **4** | **5** |
| 7 | Mtu anaweza kuzuia maambukizi ya ugonjwa kwa kuchemsha maji kwa ajili ya kumuogesha mtoto. | **1** | **2** | **3** | **4** | **5** |
| 8 | Maambukizi ya ugonjwa wa kichocho cha mkojo yanaweza kuzuilika kwa kuua konokono | **1** | **2** | **3** | **4** | **5** |
| 9 | Tiba asilia ni matibu yenye ufanisi zaidi kwenye kutibu kichocho cha mkojo. | **1** | **2** | **3** | **4** | **5** |
| 10 | Matumizi ya nguo zinazozuia maji kupenya, mtu anapokuwa kwenye vyanzo vya maji yanaweza kuzuia mtu asipate maambukizi. | **1** | **2** | **3** | **4** | **5** |
