# Supplementary material for: Prevalence and risk factors of urogenital schistosomiasis among under-fives in Mtama District in the Lindi region of Tanzania
Source: PLoS Negl Trop Dis. 2022 Apr 20;16(4):e0010381. doi: 10.1371/journal.pntd.0010381 (PMC9060350; doi:10.1371/journal.pntd.0010381)
Supplement: S1 Table — (DOCX) [file pntd.0010381.s006.docx]

**Table S1: The classification of infection intensity according to socio-demographic characteristics of the under-fives (n=385)**

| **Socio-demographics** | **Total** | ***S. haematobium* intensity** | | **p-value** |
| --- | --- | --- | --- | --- |
|  |  | **Light intensity**  **n (%)** | **Heavy intensity**  **n (%)** |  |
| **Sex of the children** |  |  | |  |
| Males | 33 | 21(63.6) | 12(36.4) | 0.026* |
| Females | 32 | 28(87.5) | 4(12.5) |  |
| **Age (months) of the children** |  |  | |  |
| 12-35 (Young children) | 14 | 13(92.9) | 1(7.1) | 0.087 |
| 36-59 (Preschoolers) | 51 | 36(70.6) | 15(29.4) |  |
| **Wards of the residency** |  |  | |  |
| Longa | 43 | 32(74.4) | 11(25.6) | 0.066 |
| Nyengedi | 13 | 10(76.9) | 3(23.1) |  |
| Nyangamara | 9 | 7(77.8) | 2(22.2) |  |

*Statistically significant (p<0.05)
